# The mitochondrial genome of the plant-pathogenic fungus *Stemphylium lycopersici* uncovers a dynamic structure due to repetitive and mobile elements

**DOI:** 10.1371/journal.pone.0185545

**Published:** 2017-10-03

**Authors:** Mario Emilio Ernesto Franco, Silvina Marianela Yanil López, Rocio Medina, César Gustavo Lucentini, Maria Inés Troncozo, Graciela Noemí Pastorino, Mario Carlos Nazareno Saparrat, Pedro Alberto Balatti

**Affiliations:** 1 Centro de Investigaciones de Fitopatología, Facultad de Ciencias Agrarias y Forestales, Universidad Nacional de La Plata - Comisión de Investigaciones Científicas de la Provincia de Buenos Aires, La Plata, Buenos Aires, Argentina; 2 Cátedra de Microbiología Agrícola, Facultad de Ciencias Agrarias y Forestales, Universidad Nacional de La Plata, La Plata, Buenos Aires, Argentina; 3 Instituto de Botánica Carlos Spegazzini, Facultad de Ciencias Naturales y Museo, Universidad Nacional de La Plata, La Plata, Buenos Aires, Argentina; 4 Instituto de Fisiología Vegetal, Facultad de Ciencias Naturales y Museo-Facultad de Ciencias Agrarias y Forestales, Universidad Nacional de La Plata - Consejo Nacional de Investigaciones Científicas y Técnicas, La Plata, Buenos Aires, Argentina; Universite Paris-Sud, FRANCE

## Abstract

*Stemphylium lycopersici* (Pleosporales) is a plant-pathogenic fungus that has been associated with a broad range of plant-hosts worldwide. It is one of the causative agents of gray leaf spot disease in tomato and pepper. The aim of this work was to characterize the mitochondrial genome of *S*. *lycopersici* CIDEFI-216, to use it to trace taxonomic relationships with other fungal taxa and to get insights into the evolutionary history of this phytopathogen. The complete mitochondrial genome was assembled into a circular double-stranded DNA molecule of 75,911 bp that harbors a set of 37 protein-coding genes, 2 rRNA genes (*rns* and *rnl*) and 28 tRNA genes, which are transcribed from both sense and antisense strands. Remarkably, its gene repertoire lacks both *atp8* and *atp9*, contains a free-standing gene for the ribosomal protein S3 (*rps3*) and includes 13 genes with homing endonuclease domains that are mostly located within its 15 group I introns. Strikingly, subunits 1 and 2 of cytochrome oxidase are encoded by a single continuous open reading frame (ORF). A comparative mitogenomic analysis revealed the large extent of structural rearrangements among representatives of Pleosporales, showing the plasticity of their mitochondrial genomes. Finally, an exhaustive phylogenetic analysis of the subphylum Pezizomycotina based on mitochondrial data reconstructed their relationships in concordance with several studies based on nuclear data. This is the first report of a mitochondrial genome belonging to a representative of the family Pleosporaceae.

## Introduction

*Stemphylium* (teleomorph: *Pleospora*) is a dematiaceous fungal genus belonging to the subphylum Pezizomycotina (Ascomycota). Species within this genus are widely distributed in nature, where they are found establishing pathogenic, saprotrophic or endophytic relationships with a broad range of plant hosts. Pathogenic representatives of this genus cause severe yield reductions and economic losses on several crops worldwide [1]. Among these phytopathogens, *Stemphylium lycopersici* has been associated with 36 plant hosts [2], it affects horticultural crops such as tomato [3] and pepper [4] as well as ornamental plants such as chrysanthemum [5] and kalanchoe [6]. Several studies mainly focused on morphological, physiological, pathogenic and genetic variation in different populations of *S*. *lycopersici* have been performed [7–10], but much research remains to be done concerning the molecular mechanisms underlying all these aspects. Regarding this, the whole-genome sequencing project of *S*. *lycopersici* CIDEFI-216 [11] might shed light on many of these issues and might also contribute to a better understanding of the evolutionary history of *Stemphylium*.

Mitochondria are semi-autonomous organelles present in most eukaryotic cells and are the main responsible of a variety of crucial cellular processes such as energy production, cell growth and apoptosis [12, 13]. They are thought to be descendants of an endosymbiotic α-proteobacterium that was engulfed by a proto-eukaryotic host cell more than one billion years ago in an event that gave rise to one of the most extreme and effective symbiotic relationship in nature. During this co-evolution, the genome of the ancient endosymbiont has undergone a remarkable size reduction since much of its genetic material have been transferred to the host or lost [14, 15].

Fungal mitochondrial DNAs are typically small, double-stranded DNA molecules with a highly compact gene organization that are present in multiple copies in each cell. The actual topology these molecules adopt *in vivo* is certainly under debate. While some authors assume a circular monomeric conformation, other authors suggest a linear concatemeric structure that, once it has been assembled, would also lead to a circular-mapping molecule. As a matter of fact, given the probability that the mitochondrial DNA of most fungal species replicates by the rolling-circle mechanism, it was also proposed that the actual genome architecture is a combination of both circular and linearly repeated. Fungal mitochondrial genomes usually harbor 14 core-genes encoding proteins involved in electron transport and oxidative phosphorylation, including the apocytochrome b (*cob*), three subunits of the cytochrome c oxidase (*cox1*, *cox2*, *cox3*), seven subunits of the reduced nicotinamide adenine dinucleotide ubiquinone oxidoreductase (*nad1*, *nad2*, *nad3*, *nad4*, *nad4L*, *nad5* and *nad6*) and three subunits of the ATP synthase (*atp6*, *atp8*, *atp9*), as well as untranslated genes of the small and large ribosomal RNA (rRNA) subunits (*rns* and *rnl*, respectively) and a set of transfer RNA (tRNA) genes. Other genes that are occasionally found within mitochondrial genomes of fungi are those that encode the ribosomal protein S3 (RPS3, coded by *rps3*) and the RNA subunit of the mitochondrial RNase P (*rnpB*). Last, but not least, fungal mitochondrial genomes also are characterized by a variable number of group I and group II introns that may carry homing endonuclease genes (HEGs) with LAGLIDADG or GIY-YIG motifs [14–18]. HEGs are selfish genetic mobile elements that encode site-specific-sequence-tolerant DNA endonucleases whose catalytic activity promotes their own propagation by the introduction DNA double-strand breaks (DSBs) into alleles lacking the endonuclease-coding sequence and by the subsequent repair of these DSBs via homologous recombination using as template the endonuclease-containing allele [19–21]. This mechanism of propagation can result in the insertion, deletion or mutation of DNA sequences [22]. Indeed, mobile introns and HEGs represent one of the major sources of variability within fungal mitochondrial genomes [16, 23, 24].

Mitochondrial genomes provide a valuable source of information to study evolutionary biology and systematics in eukaryotes. The reason for this is that they harbor conserved genes that code for proteins involved in the electron transport-oxidative phosphorylation system and that the mitochondrial DNA evolve faster than nuclear DNA, therefore, they allow the development of a robust phylogenetic analysis [25–27]. They have been successfully used in the solving of many unclear phylogenies [28, 29]. Besides, the relatively small size, circular-mapping topology and multi-copy nature of mitochondrial genomes facilitate their whole-sequencing and assembly and, thus, their study as an entity. This latter aspect is of major importance, because a complete mitochondrial genome sequence reveals the actual gene content of the organelle. Thus, it also provides additional information regarding the mitochondrial genome organization and enables the evaluation of structural rearrangements by means of comparative studies [14, 30, 31]. In addition, the high polymorphism frequently found within introns or intergenic regions of well-conserved mitochondrial genes makes these sequences useful for the study of genetic diversity among or within populations [32–34]. Mitochondrial genomes also are important in phytopathogens control, since they are the target of strobilurins, fungicides that prevent the synthesis of ATP by blocking the electron transfer at the quinol oxidation site by the cytochrome *bc*_*1*_ complex. Therefore, resistance of several fungal plant pathogens to strobilurins was associated with mutations in the mitochondrial gene *cob* [35, 36].

In this study, we assembled and annotated the mitochondrial genome of *S*. *lycopersici* CIDEFI-216, a fungus isolated from a tomato plant with symptoms of gray spot disease. We have thoroughly characterized it in terms of its gene content and organization, codon usage and by the occurrence of repetitive elements. We have also explored the evolutionary dynamics of the mitochondrial genomes of Dothideomycetes by a comparative mitogenomic approach. Finally, we carried out an extensive phylogenetic analysis aimed to infer the evolutionary relationships among 82 Pezizomycotina species, as revealed by the concatenated amino-acid sequences of 12 conserved mitochondrial-encoded proteins. This work describes mitochondrial genome of *S*. *lycopersici* CIDEFI-216, which, to our knowledge, is the first report for a member of the family Pleosporaceae (Pleosporales, Ascomycota).

## Materials and methods

### Sequence data and mitochondrial genome de novo assembly

The mitochondrial genome of *S*. *lycopersici* CIDEFI-216 was sequenced on an Illumina Hiseq 2000 platform (Illumina, San Diego, CA) using a 100 bp paired-end approach (insert size of 300 bp) in the course of the *S*. *lycopersici* CIDEFI-216 whole genome shotgun project (BioProject: PRJNA274742, BioSample: SAMN03332054) [11]. Even though the data were originated from total genomic DNA, the mitochondrial fraction of the sequence reads was expected to be over-represented as most eukaryotic cells contain multiple mitochondria, each with several copies of the genome. Hence, only a subset of 4,000,000 sequence reads (Sequence Read Archive: SRX872418) were used for the mitochondrial genome assembly. Low-quality bases at the ends of the sequence reads were trimmed off and the mitochondrial genome was assembled with the Geneious 9.1.2 *de novo* assembler [37] using low sensitivity, enabling the option to circularize contigs with matching ends. This procedure was repeated using different sets of 4,000,000 sequence reads in order to verify the recovery of the same mitochondrial DNA molecule.

### Mitochondrial genome annotation

Gene annotation was performed with MFannot (http://megasun.bch.umontreal.ca/cgi-bin/mfannot/mfannotInterface.pl) using the NCBI translation Table 4 (The Mold, Protozoan, and Coelenterate Mitochondrial Code and the Mycoplasma/Spiroplasma Code). MFannot predictions were individually checked to confirm genes boundaries as well as intron-exon boundaries by aligning the predicted genes with their orthologous in closely-related fungal species. Transfer-RNA annotations were further confirmed by tRNAscan-SE 1.21 [38]. Functional annotation of the predicted open reading frames (ORFs) was complemented with Blast2GO Basic [39]. A physical map of the mitochondrial genome was created with OrganellarGenomeDRAW (OGDRAW) v 1.2 [40] and modified with Inkscape 0.91 (http://inkscape.org/). The mitochondrial genome of *S*. *lycopersici* CIDEFI-216 has been deposited at DDBJ/EMBL/GenBank under the accession number KX453765.

### Identification of repetitive sequences

Repetitive sequences were identified using the Vmatch software (http://www.vmatch.de/). Both direct and inverted repeats (palindromes) were computed by extending a seed of 30 nucleotides in length in both directions allowing for matches, mismatches, insertions and deletions with an X-drop value for edit distance extension of 3. The screening was restricted to repetitive sequences of at least 30 nucleotides in length and with a minimum match identity of 80%. Following the analysis, overlapped repeats were manually deleted. A physical map of the repetitive sequences in the mitochondrial genome of *S*. *lycopersici* CIDEFI-216 was created with Circos [41] and modified with Inkscape 0.91 (http://inkscape.org/).

### Comparative mitogenomics

The extent of structural rearrangements in the mitochondrial genomes of *S*. *lycopersici* CIDEFI-216 and the Pleosporales species whose mitochondrial genomes were publicly available in the NCBI Genome database (https://www.ncbi.nlm.nih.gov/genome/browse/?report=5) by the end of December 2016, namely *Didymella pinodes* (GenBank accession number KT946597), *Parastagonospora nodorum* (GenBank accession number EU053989; [42]) and *Shiraia bambusicola* (GenBank accession number KM382246; [43]), was assessed by means of a multiple alignment using Mauve [44]. For comparative purposes, the first residue of the large ribosomal subunit was arbitrary set as the origin of all mitochondrial sequences. Mauve was run using the default settings and the backbone output was modified with Inkscape 0.91 (http://inkscape.org/).

### Phylogenetic analysis

The phylogeny of the subphylum Pezizomycotina was inferred by the concatenated amino acid sequences of 12 proteins encoded by the conserved mitochondrial genes involved in the oxidative phosphorylation and electron transport, namely: *atp6*, *cob*, cox1, cox2, cox3, *nad1*, *nad2*, *nad3*, *nad4*, *nad4L*, *nad5* and *nad6*. This data set excluded the products of the genes *atp8* and *atp9* as they are not shared across the entire subphylum. The analysis comprised all Pezizomycotina species whose mitochondrial genome was publicly available in the NCBI Genome database (https://www.ncbi.nlm.nih.gov/genome/browse/?report=5) by the end of December 2016 and contained the 12 conserved mitochondrial genes mentioned above. Following this criterion, a representative of the subphylum Taphrinomycotina (*Pneumocystis carinii)* and two representatives of the subphylum Saccharomycotina (*Yarrowia lipolytica* and *Komagataella pastoris)* were selected as outgroups. Hence, the analysis involved a total of 85 species representing 54 genera. Amino acid sequences were concatenated and aligned with MUSCLE [45] in Geneious 9.1.2 [37]. The resultant alignment was automatically curated with Gblocks 0.91b [46] using default settings except for the minimum length of a block which was set to 2. The selection of the best-fit model of protein evolution was carried out with ProtTest 3.2 [47] using the Akaike Information Criterion [48]. The phylogeny was reconstructed using maximum-likelihood (best-fit model: LG+I+G+F) in PhyML 3.0 [49] and Bayesian inference (best-fit available model: LG+I+G) in MrBayes 3.2 [50]. The support of the maximum-likelihood tree was estimated through 1000 bootstrap replicates [51]. For the Bayesian analysis, two independent Metropolis-coupled Markov chain Monte Carlo (MCMCMC) with four chains (one cold and three heat) were performed through one million of generations. Trees in each chain were sampled every 250th generation, the first 25% of trees were removed as burn-in and posterior probabilities were determined from the remaining trees. Phylogenetic trees were edited in FigTree v1.4.2 (http://tree.bio.ed.ac.uk/software/figtree/) and Inkscape 0.91 (http://inkscape.org/).

## Results and discussion

### General features

**Fig 1:**
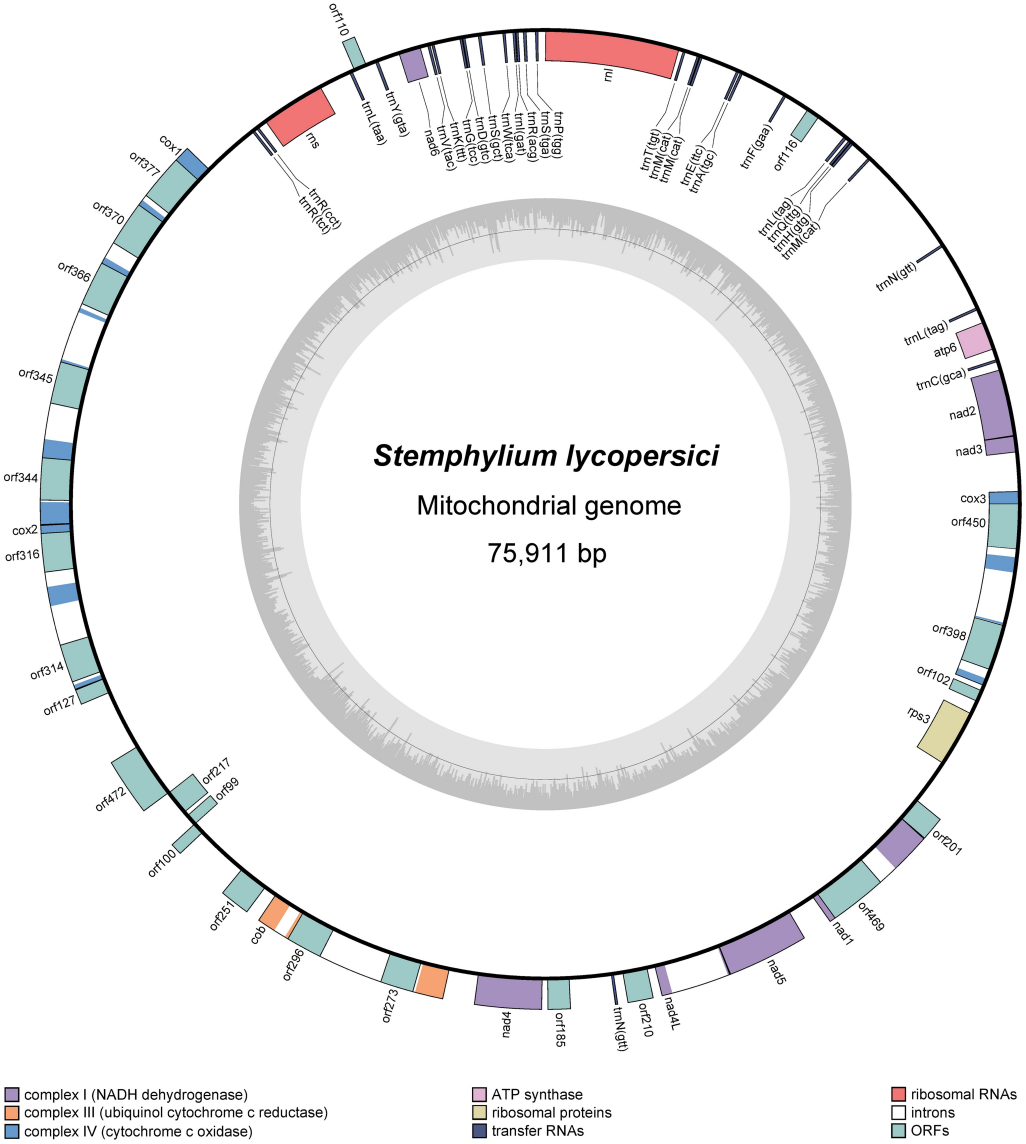
Circular map of the mitochondrial genome of *S*. *lycopersici* CIDEFI-216. Genes are represented by boxes, which are colored on the basis of their function. The inner boxes represent genes that are transcribed in a clockwise direction and the outer boxes show genes transcribed in a counterclockwise direction. The internal plot shows the GC content, with the black line marking 50%. The exact positions of the genes are indicated in Table 2. The physical map was created with OGDRAW.

The mitochondrial genome of *S*. *lycopersici* CIDEFI-216 was *de novo* assembled into a single, circular, double-stranded DNA molecule of 75,911 bp, with mean coverage of 117.74 (minimum coverage of 24; maximum coverage of 200). It contains a set of 37 protein-coding genes, 2 rRNA genes (*rns* and *rnl)* and 28 tRNA genes. Genes are transcribed from both DNA strands, whose 62.9% correspond to non-coding DNA. With a GC content of 29.6%, it reaches the upper limit of the normal values for fungal mitochondria (24.4 ± 7.3%, estimated from all fungal mitochondrial genomes deposited in the Genome database of the NCBI by January 2016). The GC content of the protein-coding genes is quite similar to the overall GC content (29.1%), however considerable deviations are found in the GC content of rRNAs and tRNA genes, reaching 35% and 39%, respectively (Fig 1, Tables 1 and 2).

**Table 1:** General features of the mitochondrial genome of *S*. *lycopersici* CIDEFI-216.

Feature	Value
Genome size (bp)	75,911
GC content (%)	29.6
No. of protein-coding genes	37
GC content of protein-coding genes (%)	29.1
No. of rRNAs	2
GC content of rRNAs genes (%)	35.0
No. of tRNAs	28
GC content of tRNAs genes (%)	39.6
No. of introns	15
Intragenic regions (%)	30.2
Intergenic regions (%)	32.7
Repetitive regions (%)	5.6

**Table 2:** Gene organization of the mitochondrial genome of *S*. *lycopersici* CIDEFI-216.

Gene	Start position	Stop position	Lenght (nt)	Orientation
*rnl*	1	3,459	3,459	forward
*trnT(tgt)*	3,559	3,629	71	forward
*trnM(cat)*	3,937	4,007	71	forward
*trnM(cat)*	4,013	4,085	73	forward
*trnE(ttc)*	5,014	5,086	73	forward
*trnA(tgc)*	5,119	5,190	72	forward
*trnF(gaa)*	6,366	6,438	73	forward
*orf116*	7,077	7,427	351	forward
*trnL(tag)*	8,240	8,322	83	forward
*trnQ(ttg)*	8,393	8,464	72	forward
*trnH(gtg)*	8,470	8,543	74	forward
*trnM(cat)*	9,059	9,130	72	forward
*trnN(gtt)*	11,959	12,029	71	forward
*trnL(tag)*	13,797	13,869	73	forward
*atp6*	14,170	14,943	774	forward
*trnC(gca)*	15,216	15,287	72	forward
*nad2*	15,490	17,220	1,731	forward
*nad3*	17,221	17,643	423	forward
*cox3*	18,638	23,719	5,082	forward
*orf450*	18,971	20,323	1,353	forward
*orf398*	22,090	23,286	1,197	forward
*orf102*	23,847	24,155	309	forward
*orf490*	24,489	25,961	1,473	forward
*orf201*	27,104	27,709	606	reverse
*nad1*	27,709	30,731	3,023	reverse
*orf469*	29,178	30,587	1,410	reverse
*nad5*	31,440	33,431	1,992	reverse
*nad4L*	33,431	35,147	1,717	reverse
*orf210*	35,347	35,979	633	reverse
*trnN(gtt)*	36,210	36,280	71	reverse
*orf185*	37,345	37,902	558	reverse
*nad4*	38,034	39,671	1,638	reverse
*cob*	40,418	45,259	4,842	reverse
*orf273*	41,156	41,977	822	reverse
*orf296*	43,538	44,428	891	reverse
*orf251*	45,585	46,340	756	reverse
*orf100*	47,708	48,010	303	reverse
*orf99*	47,994	48,293	300	forward
*orf217*	48,394	49,047	654	forward
*orf472*	49,044	50,462	1,419	reverse
*orf127*	52,012	52,395	384	reverse
*cox2* ^a^	52,395	56,445	4,051	reverse
*orf314*	52,587	53,531	945	reverse
*orf316*	55,300	56,250	951	reverse
*cox1* ^a^	56,446	66,406	9,961	reverse
*orf344*	57,032	58,066	1,035	reverse
*orf345*	59,369	60,406	1,038	reverse
*orf366*	61,869	62,969	1,101	reverse
*orf370*	63,510	64,622	1,113	reverse
*orf377*	64,886	66,019	1,134	reverse
*trnR(tct)*	67,861	67,931	71	forward
*trnR(cct)*	67,995	68,065	71	forward
*rns*	68,284	69,953	1,670	forward
*orf110*	70,908	71,240	333	reverse
*trnY(gta)*	71,478	71,562	85	forward
*nad6*	72,116	72,691	576	forward
*trnV(tac)*	72,876	72,948	73	forward
*trnK(ttt)*	72,981	73,052	72	forward
*trnG(tcc)*	73,711	73,783	73	forward
*trnD(gtc)*	73,786	73,857	72	forward
*trnS(gct)*	74,188	74,267	80	forward
*trnW(tca)*	74,820	74,891	72	forward
*trnI(gat)*	75,077	75,148	72	forward
*trnR(acg)*	75,153	75,224	72	forward
*trnS(tga)*	75,345	75,429	85	forward
*trnP(tgg)*	75,655	75,727	73	forward

^a^ Both *cox1* and *cox2* are encoded by a single continuous open reading frame.

### Protein-coding genes

The structural and functional annotation of the mitochondrial genome of *S*. *lycopersici* CIDEFI-216 led to the identification of 37 protein-coding genes. Twelve of them were related to the electron transport and oxidative phosphorylation system, and included *cob*, *cox1*, *cox2*, *cox3*, *nad1*, *nad2*, *nad3*, *nad4*, *nad4L*, *nad5*, *nad6* and *atp6*, which are considered core protein-coding genes. We also found a gene homologous to *rps3*, 13 genes homologues to GIY-YIG or LAGLIDADG homing endonucleases, a gene with homology to an N-acetyltransferase and 10 protein-coding genes with unknown function (Table 3 and S1 Table). All genes are transcribed in sense and anti-sense directions. Protein-coding genes account for about 57.81% of the mitochondrial genome, whilst its coding sequences represent the 44.69%. The observed difference is attributed to the presence of 15 group I introns, many of them harboring ORFs that mostly encode GIY-YIG or LAGLIDADG homing endonucleases. The core protein-coding genes exhibited some notable features. Undoubtedly, the most striking feature was the fusion between *cox1* and *cox2*, as suggested by its gene architecture. This putative *cox1/2* gene fusion was riddled with introns, accounting for nearly half of the introns found in the mitochondrial genome. Another remarkable feature was the absence of genes encoding the subunits 8 and 9 of the ATP synthase (*atp8* and *atp9*, respectively), since both genes are conserved among fungal mitochondria [16, 31, 52]. The aforementioned characteristics are further discussed below.

**Table 3:** Characteristics of the protein-coding genes of the mitochondrial genome of *S*. *lycopersici* CIDEFI-216.

Gene	Introns	Coding sequence density (%)	Start Codon	Stop Codon	Product	Length (aa)
***atp6***	0	100	ATG	TAG	ATP synthase F0 subunit a	257
***cob***	2	23.9	ATG	TAG	Apocytochrome b	385
***cox1*** ^1^	6	16.6	ATG	TGT ^2^	Cytochrome c oxidase subunit 1	551
***cox2*** ^1^	2	17.7	GAT ^3^	TAA	Cytochrome c oxidase subunit 2	238
***cox3***	3	15.9	ATG	TAG	Cytochrome c oxidase subunit 3	269
***nad1***	1	36.9	ATG	TAA	NADH dehydrogenase subunit 1	371
***nad2***	0	100	ATG	TAA	NADH dehydrogenase subunit 2	576
***nad3***	0	100	ATG	TAG	NADH dehydrogenase subunit 3	140
***nad4***	0	100	ATG	TAA	NADH dehydrogenase subunit 4	545
***nad4L***	1	100	ATG	TAA	NADH dehydrogenase subunit 4L	89
***nad5***	0	100	ATG	TAA	NADH dehydrogenase subunit 5	663
***nad6***	0	100	ATG	TAA	NADH dehydrogenase subunit 6	191
*rps3*	0	100	ATG	TAA	Ribosomal protein S3	490
*orf99*	0	100	ATG	TAA	Hypothetical protein	99
*orf100*	0	100	ATG	TAG	Hypothetical protein	100
*orf102*	0	100	ATG	TAA	LAGLIDADG domain-containing protein	102
*orf110*	0	100	ATG	TAA	Hypothetical protein	110
*orf116*	0	100	ATG	TAA	Hypothetical protein	116
*orf127*	0	100	ATG	TAA	Hypothetical protein	127
*orf185*	0	100	ATG	TAA	Hypothetical protein	185
*orf201*	0	100	ATG	TAG	GIY-YIG domain-containing protein	201
*orf210*	0	100	ATG	TAA	Hypothetical protein	210
*orf217*	0	100	ATG	TAA	N-acetyltransferase domain-containing protein	217
*orf251*	0	100	ATG	TAA	Hypothetical protein	251
*orf273* ^4^	0	100	ATG	TAA	LAGLIDADG domain-containing protein	273
*orf296* ^4^	0	100	AAA ^3^	TAG	LAGLIDADG domain-containing protein	296
*orf314* ^4^	0	100	ATG	TAA	GIY-YIG domain-containing protein	314
*orf316* ^4^	0	100	AAA ^3^	TAA	GIY-YIG domain-containing protein	316
*orf344* ^4^	0	100	CAA ^3^	TAA	LAGLIDADG domain-containing protein	344
*orf345* ^4^	0	100	AAA ^3^	TAG	Hypothetical protein	345
*orf366* ^4^	0	100	AAA ^3^	TAA	LAGLIDADG domain-containing protein	366
*orf370* ^4^	0	100	GGA ^3^	TAA	GIY-YIG domain-containing protein	370
*orf377* ^4^	0	100	GAA ^3^	TAG	LAGLIDADG domain-containing protein	377
*orf398* ^4^	0	100	ATG	TAG	LAGLIDADG domain-containing protein	398
*orf450* ^4^	0	100	AAA ^3^	TAA	LAGLIDADG domain-containing protein	450
*orf469* ^4^	0	100	TGT ^3^	TAA	GIY-YIG domain-containing protein	469
*orf472*	0	100	ATG	TAA	Hypothetical protein	472

The conserved mitochondrial genes involved in the electron transport and oxidative phosphorylation are in bold type letter.

^1^ Both *cox1* and *cox2* seems to be encoded by a single continuous open reading frame.

^2^ Stop codon not determined. Possibly, the ORF is co-translated with the downstream exon(s) and then further processed to yield the corresponding mature proteins.

^3^ Start codon not determined. Possibly, the ORF is co-translated with the upstream exon(s) and then further processed to yield the corresponding mature proteins.

^4^ Gene located within an intron.

### ATP synthase subunits 8 and 9 are encoded by the nuclear genome

In billon of years of co-evolution with its host-cell and specialization into an energy-producing organelle, the ancestor of mitochondria lost most of its genetic material. This might have led to the elimination of genes whose function either become obsolete or were relieved by the corresponding nuclear-gene counterpart. Indeed, it has been shown that most of its genetic material has been migrating to the nucleus as part of a process that is still ongoing. This is such that almost the entire mitochondrial proteome is actually encoded by nuclear-genes, synthesized in the cytosol and imported into the mitochondria by various different mechanisms, among which is the recognition of a mitochondrial-targeting peptide attached to the N-terminus of the corresponding pre-protein [15, 53].

The fungal mitochondrial genomes sequenced so far generally has a standard set of 14 protein-coding-genes that includes *atp6*, *atp8*, *atp9*, *cob*, *cox1*, *cox2*, cox3, *nad1*, *nad2*, *nad3*, *nad4*, *nad4L*, *nad5* and *nad6* [14, 31, 52]. There are, however, exceptions, as is attested by the absence of the *atp9* gene in *Podospora anserina* (Sordariales) [54]; of *atp8* and *atp9* genes in the Pleosporales *D*. *pinodes* (KT946597), *P*. *nodorum* [42] and *S*. *bambusicola* [43]; of *cox3* and *nad6* genes in *Acremonium implicatum* (Hypocreales) [55]; and the lack of *nad1*, *nad2*, *nad3*, *nad4*, *nad4L*, *nad5* and *nad6* genes in the mitochondrial genome of several yeasts [56–60]. We found that the mitochondrial genome of *S*. *lycopersici* CIDEFI-216 lacks both *atp8* and *atp9* genes, which is in agreement with observations made in other representatives of Pleosporales. Interestingly, these two genes are present in the mitochondrial genome of the Capnodiales *Zasmidium cellare* [61] and *Zymoseptoria tritici* [62], which suggests that these genes might have been lost at some point after the divergence between Dothideomycetidae and Pleosporomycetidae (Dothideomycetes). We searched for the presence of these genes in the nuclear of genome *S*. *lycopersici* CIDEFI-216 by doing BLAST [63] searches using the *atp8* and *atp9* nucleotide and amino-acid sequences of the closest related *Z*. *cellare* and *Z*. *tritici* as queries in a database composed by the 214 scaffolds of the draft genome of *S*. *lycopersici* CIDEFI-216 and its predicted proteome (GenBank accession number LGLR00000000.1). We found a gene highly homologous to *atp8* (Locus tag TW65_04673) in the scaffold 66 (GenBank accession number LGLR01000223) and another one highly homologous to *atp9* (Locus tag TW65_03276) in the scaffold 40 (GenBank accession number LGLR01000197). Both nuclear-genes were larger than their mitochondrial homologues, leaving a flanking sequence at their 3’-ends (N-terminus of each protein). The refinement of the alignment (ClustalW, default settings) to the homologous regions yielded a pairwise identity of 66.2% and 70.7% at the nucleotide level and 64.7% and 70.2% at the amino-acid level for *atp8* and *atp9*, respectively (S1 Fig). Furthermore, TargetP 1.1 [64] predicted the presence of N-terminal mitochondrial-targeting peptides in the putative products of *atp8* and *atp9* nuclear homologues (S2 Table).

### A putative gene fusion between *cox1* and *cox2*

Cytochrome-c oxidase (EC 1.9.3.1) is a large oligomeric enzymatic complex that mediates the final step of the respiratory chain. In eukaryotes, this complex is composed by three large subunits, the cytochrome-c oxidase subunits I, II and III (COX1, COX2 and COX3, respectively), which comprise the catalytic center of the enzymatic complex together with a variable number of nuclear-encoded polypeptides [65]. Generally, COX1, COX2 and COX3 are encoded by three independent genes (*cox1*, *cox2* and *cox3*, respectively) that are located in the mitochondrial genome, with few exceptions in *cox2* and *cox3* [14, 66]. Given the important biological process that they fulfill, they are highly conserved even among distantly-related species, because of this, *cox1* has been used as a molecular barcode in several taxonomic and evolutionary studies [67, 68].

The predicted gene structure of the mitochondrial genome of *S*. *lycopersici* CIDEFI-216 presented an unusual gene organization of two subunits of the cytochrome-c oxidase. Specifically, COX1 and COX2 are encoded by a single continuous ORF that is 2,370 bp long and begin and terminate with the canonical start and stop codons ATG and TAA, respectively. This so-called cox1/2 ORF contains the coding sequences of both COX1 and COX2 in the same frame lacking a canonical stop codon between them, which raises the possibility of a fusion transcript and/or protein. In this hypothetical transcript, COX1 sequence is extended to nucleotide 1,653, where the COX2 sequence begins.

**Fig 2:**
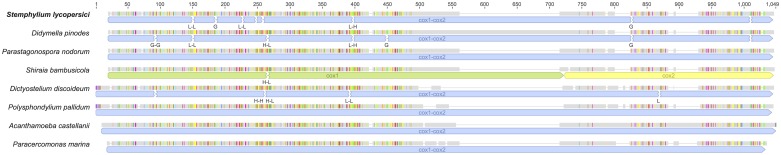
Schematic sequence alignment of COX1 and COX2 with two different gene configurations. Amino-acid identities are highlighted with colors. COX1, COX2 and COX1/2 regions are indicated by green, yellow and blue boxes. Intron positions are represented by a discontinuity in the boxes. Any recognizable homing endonuclease domain, LAGLIDADG domain and/or GIY-YIG domain found within the hypothetical proteins encoded by the intronic ORFs are depicted by the letters H, L and G, respectively. The amino-acid sequences were aligned using MUSCLE with the default settings in Geneious 9.1.2. The detailed alignment is found in S2 Fig.

This peculiar gene organization also was found in other representatives of the Pleosporales such as *D*. *pinodes* (KT946597) and *P*. *nodorum* [42]. In *S*. *bambusicola*, however, the aforementioned subunits are encoded by a pair of genes that are adjacent with no intergenic or overlapping nucleotides [43]. Similarly, a cox1/2 ORF was found in the protozoans *Acanthamoeba castellanii* [69, 70], *Dictyostelium discoideum* [71] and *Polysphondylium pallidum* (AY700145), and the cercozoan *Paracercomonas marina* (KP165385), which, in spite of the large evolutionary distance to fungi from Pleosporales, are placed near the base of radiation that leads to the animals and fungi [25, 72]. Indeed, conserved homologous blocks belonging to both subunits can be clearly identified within the multiple sequence alignment of the putative COX1/2 protein with homologous sequences from Pleosporales and the above-mentioned protists (Fig 2 and S2 Fig).

Such gene architecture was first reported by Burger et al. in 1995, describing the mitochondrial genome of *A*. *castellanii* [69]. A year later, Lonergan and Gray proved that, in *A*. *castellanii*, this ORF is transcribed into a large polycistronic mRNA that upon translation give rise to two products [70]. Next, Pellizzari et al found in the mitochondrial genome of *D*. *discoideum* evidences of a similar *cox1/2* transcript [71]. However, whether COX1 and COX2 are synthesized separately or as a fused protein in *S*. *lycopersici* remains unclear. These subunits might be encoded by the nuclear genome, whereas the mitochondrial cox1/2 represents a dysfunctional copy. However, we were unable to find the coding sequences of both subunits in the nuclear genome of *S*. *lycopersici* CIDEFI-216 by doing BLAST searches against its predicted ORFeome and proteome. So, it appears that the mitochondrial *cox1/2* locus is unique and, thus, has to be functional. The *cox1/2* transcript may also contain an unusual stop codon immediately after the cox1 CDS, but this is rather unlikely in view of the codon usage found in the conserved protein-coding genes of this mitochondrial genome, which will be discussed in a later section. Finally, we could not rule out that the *cox1/2* primary-transcript might be edited so as to include a termination codon in the mature mRNA, or be endonucleolytically processed to yield the two mature mRNAs. Future studies should be aimed at answering this question.

The *cox1/2* gene sequence has a length of 14,012 nucleotides, contains 8 group I introns, 5 of which harbor ORFs that might encode homing endonucleases, as it could be expected by their predicted LAGLIDADG or GIY-YIG domains. Interestingly, the location of some of these introns is well-conserved, even among distantly-related species (Fig 2 and S2 Fig). So, it appears that c*ox1* is a hotspot for insertion of group I introns [73, 74]. Whether group I introns encode homing endonucleases functionally active or are just footprints of previous insertional events, they provide valuable information to trace evolutionary history. Interestingly, there is substantial evidence that group I intron-encoded homing endonucleases are involved in processes such as genome rearrangements and horizontal gene transfer [75–77], suggesting these two possible scenarios that could explain the observed gene fusions between *cox1* and *cox2* in quite distantly-related taxa. The gene fusion might have arisen independently in different lineages due to the exon shuffling promoted by the homing endonuclease-mediated mobility of group I introns. Alternatively, the fusion might be the result of a horizontal gene transfer event that might have occurred at some point during the evolution of Dothideomycetes and Protozoans.

### A free-standing *rps3* and an intronless *rnl*

**Fig 3:**
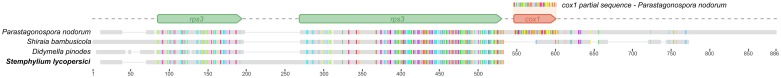
Schematic sequence alignment of RPS3 from Pleosporales species. It can be clearly seen conserved blocks of homology to RPS3 shared between the analyzed Pleosporales species. Regions of RPS3 that are conserved among Pleosporales are indicated with green boxes. The region homologous to COX1 particularly found in *P*. *nodorum* is indicated with a red box. The amino-acid sequences were aligned using MUSCLE with the default settings in Geneious 9.1.2. The detailed alignment is found in S3 Fig.

The mitochondrial genome of *S*. *lycopersici* CIDEFI-216 contains a free-standing gene that is 1,473 bp long that code for a 490 amino-acids protein with partial homology to several fungal RPS3, a key protein component of the ribosome, essential for protein translation. *Rps3* is widely distributed throughout all life kingdoms and is certainly diverse in sequence, structure and location [78, 79]. The first mitochondrial ribosomal protein described in fungi, specifically in yeast, was VAR1 that is coded by the free-standing gene *var1* [80]. Later, Burke and RajBhandary found an intronic ORF for the putative ribosomal protein S3 within the *rnl* U11 group I intron (also known as mL2449 group I intron) in *Neurospora crassa* (Sordariales) [81]. Currently, the general consensus is that RPS3, VAR1 and S5 are homologs [78, 79]. Although the longest protein orthologs usually contain partial sequences with no significant homology to any known protein, sometimes they contain partial sequences homologous to known proteins such as in some *Ophiostoma* spp. (Ophiostomatales), where *rps3* is fused to homing endonuclease domains [82]. Furthermore, while in *P*. *nodorum*, *rps3* also contains an internal sequence highly homologous to a partial sequence of *cox1* [42, 79], in *Sphaeronemella fimicola* (Microascales), *rps3* includes an in-frame micro-satellite insertion that results in the presence of 16 consecutive alanines [79]. In light of the findings described above, it is evident that *rps3* has a rather complex evolutionary history, which makes more interesting their sequence analysis among representatives of Ascomycota. Therefore, we used all the available mitochondrial genomes in the GenBank (Accessed in April 2017) and, when necessary, annotated them as described previously. We found that, in Saccharomycotina, the ribosomal protein S3 is coded by a free-standing gene and, on the other hand, the *rnl* usually contains an intron that in some cases harbor an ORF homologous to homing endonucleases. In Taphrinomycotina, *rps3* exists as a free-standing gene, through no intron was found in *rnl*. Within Pezizomycotina, *rps3* was found within the mL2449 group I intron of the *rnl* in members of Eurotiomycetes and Sordariomycetes. However, in Leotiomycetes and Lecanoromycetes, *rps3* exists either as a free-standing gene or within the mL2449 group I intron of *rnl*. Surprisingly, Dothideomycetes contains an intronless *rnl* and, while the mitochondrial genomes of *Z*. *cellare* and *Z*. *tritici* (Capnodiales) lack the *rps3*, the ones of *D*. *pinodes*, *S*. *bambusicola*, *S*. *lycopersici* and *P*. *nodorum* (Pleosporales) contain a free-standing *rps3*, that only in the latter is fused with a partial sequence of *cox1* (Fig 3 and S3 Fig). Probably, these gain-loss events that led to the configurations described might have been driven by the presence of an essential gene such as *rps3* within the mL2449 group I intron that ensured the maintenance of this neutral element, which otherwise might have eventually been lost, but also by the homing endonuclease-mediated mobility that might be responsible for the relocation of *rps3* from its original position (whichever it was) to the mL2449 group I intron and/or its settlement as a free-standing gene. This is further supported by the presence of LAGLIDADG homing endonuclease-*rps3* fusions within the *rnl* Group I Intron in some *Ophiostoma* spp. [82] as well as by the fact that the *rps3* of *P*. *nodorum* presents a footprint of a previous insertion of the *cox1* [32, 79], a gene that is usually invaded by these mobile genetic elements [75– 77]. It is worth mentioning that we did not find a *rps3* counterpart in the nuclear genome of any representative of Dothideomycetes.

### Fertile ground for introns and homing endonucleases

Group I and group II introns are ribozymes that catalyze their splicing from a precursor RNA, restoring the translational reading frame and generating, in this way, a functional product. This selfish DNA is frequently present in mitochondrial genomes, though while group I introns are abundant in fungal mitochondrial genomes, group II introns are predominant in those of plants [17]. Both types of introns propagate themselves as mobile genetic elements, through different mechanisms. Mobility of group I intron is due to the activity of homing of endonucleases, whose coding sequences reside in non-critical sequences of the intron. On the other hand, mobility of group II introns relies on a mechanism known as retro-homing, which is catalyzed by an intron-encoded protein with reverse-transcriptase and maturase domains, and sometimes an endonuclease domain [83]. In fact, homing endonucleases themselves can be mobile elements, moving independently from their host intron, like the free-standing homing endonucleases, which are frequent within phage genomes [21]. It is intriguing how such genes settled within unspoiled introns to yield these composite mobile elements. Whatever the reason, this co-evolution left several footprints along genomes over eons and, hence, these genetic fossils are a valuable source of information for evolutionary studies.

In the mitochondrial genome of *S*. *lycopersici* CIDEFI-216 we found 15 introns, all corresponding to group I, many of them harboring ORFs that mostly encode GIY-YIG or LAGLIDADG domain-containing proteins. All introns were located within conserved genes involved in the the electron transport and the oxidative phosphorylation system, which, ordered by frequency, are: *cox1/2* (8), *cox3* (3), *cob* (2), *nad1* (1) and *nad4L* (1) (Table 3). As it was mentioned before, *cox1* is a common target for the insertion of introns in fungi and other eukaryotes [75–77]. The mitochondrial genome of *S*. *lycopersici* CIDEFI-216 also contains 13 genes, whose hypothetical proteins present domains characteristic of homing endonucleases (S1 Table). While 8 of the hypothetical proteins carried the LAGLIDADG domain, 5 of them carried the GIY-YIG domain. Most of the homing endonuclease genes in the mitochondrial genome of *S*. *lycopersici* CIDEFI-216 are placed within group I introns, however two of them exist as free-standing genes (*orf102* for the LAGLIDADG domain-containing protein and *orf201* for the GIY-YIG domain-containing protein). Interestingly, they account for more than one-third of the total protein-coding genes of the mitochondrial genome.

It is important to compare intron positions within the *cox1/2* (*cox1* and *cox2*) sequence from the mentioned Dothideomycetes, slim molds and cercozoan representatives (Fig 2). Among the known sequences of *cox1/2* in Dothideomycetes, *S*. *lycopersici* has the highest number of introns (8 introns harboring 5 homing endonucleases), followed by *D*. *pinodes* (7 introns but 6 homing endonucleases) (GenBank accession number KT946597). Furthermore, while *S*. *bambusicola* has a *cox1* that contains a single intron (1 homing endonuclease), *cox2* lacks introns [43]. On the other hand, *cox1/2* in *P*. *nodorum* and *cox1* and *cox2* in *Z*. *cellare* and *Z*. *tritici* (Capnodiales) lack introns [42, 61, 62]. In the distantly-related slime mold *D*. *discoideum*, *cox1/2* contains 4 introns with 4 homing endonucleases (two in the same intron) [71], while the protozoans *A*. *castellanii* [69] and *P*. *pallidum* [70], and the cercozoan *P*. *marina* (GenBank accession number KP165385) lack introns. The presence of an intron in the same position in a distantly-related organism suggests that such structural feature could be the result of an ancestral event of insertion. Considering this, the first intron of *D*. *pinodes* and the first intron of *D*. *discoideum* might have a common ancestral origin, though the latter lost their intron-mobility enzyme. However, all the other studied organisms lost this intron at some point of their evolutionary history. Hence, we hypothesized that the remaining introns might have arisen from more recent insertional events. Interestingly, the position of the third intron of *D*. *pinodes* and the single intron within *cox1* of *S*. *bambusicola* correlates with a slight shift, regarding the position of the second intron of *D*. *discoideum*. Similarly, the sixth intron of *S*. *lycopersici* and the fourth intron of *D*. *pinodes* with the third intron of *D*. *discoideum* are also correlated. We might have detected cases of lost-and-gain events, not only because of the aforementioned slight variation in introns position between these distantly-related organisms, but also because of the low level of amino-acid identity shared by their mobility proteins. We might speculate that the earliest insertional event gave rise to the seventh intron in *S*. *lycopersici* and the sixth intron in *D*. *pinodes*, since both of them contains LAGLIDADG domain-containing homing endonucleases with a high level of pairwise identity at the amino-acid level (Pairwise% Identity: 87.9%; Pairwise% Positive (BLSM62): 94.8%).

### tRNAs genes and codon bias

The mitochondrial genome of *S*. *lycopersici* CIDEFI-216 has a set of 28 tRNA genes, including all those needed to decode the codons of each of the 20 standard amino-acids required for the synthesis of the predicted mitochondrial-encoded proteins (Table 4). As a result of this, mitochondria might not need to import any nuclear-encoded tRNA from the cytosol to synthesize their proteins, as it occurs in many fungal, plant and animal mitochondria [14–16]. The tRNA gene repertoire of *S*. *lycopersici* CIDEFI-216 includes 3 copies of t*rnM(cat)*, 2 of *trnN(gtt)* and 2 of *trnL(tag)*. It also has tRNA genes with different anticodons that incorporate the same amino-acid, namely: *trnR(acg)*, *trnR(cct)* and *trnR(tct)* for Arginine; *trnE(ttc)* and *trnE(ttc)* for Glutamine; and *trnS(tga)* and *trnS(gct)* for Serine. Most tRNA genes are transcribed in a clockwise direction from a region spanning upstream of *rns* and downstream of *atp6*. The sole exception is one of the two copies of *trnN(gtt)*, which is located considerably farther downstream from the tRNA-rich region and is transcribed in counterclockwise direction (Fig 1).

**Table 4:** tRNAs within the mitochondrial genome of *S*. *lycopersici* CIDEFI-216.

Amino Acid	Anticodon	Number
Ala	UGC	1
Arg	ACG	1
Arg	CCU	1
Arg	UCU	1
Asn	GUU	2
Asp	GUC	1
Cys	GCA	1
Gln	UUG	1
Glu	UUC	1
Gly	UCC	1
His	GUG	1
Ile	GAU	1
Leu	UAA	1
Leu	UAG	2
Lys	UUU	1
Met	CAU	3
Phe	GAA	1
Pro	UGG	1
Ser	GCU	1
Ser	UGA	1
Thr	UGU	1
Trp	UCA	1
Tyr	GUA	1
Val	UAC	1

While we were trying to identify the translational start and stop codons of the mitochondrial genes of *S*. *lycopersici* CIDEFI-216, a number of rather unusual translational signals drew our attention. Specifically, the identification of a putative termination codon TGT of COX1, which was followed by a hypothetical initiation codon TGT of COX2, in addition to the presumptive initiation codons AAA, CAA, GGA, GAA and TGT used for many homing endonucleases (Table 3). The existence of such signals in the mitochondrial genome of *S*. *lycopersici* CIDEFI-216 is very unlikely considering the genetic code that is used in closely-related species [42, 43, 61, 62], but also because the only difference within conserved genes occurred with COX1 and COX2, which has already been discussed in detail. A possible explanation for this observation might be the co-translation of these ORFs carrying suspicious initiation codons alongside with their upstream exon(s), which later should be processed in order to obtain the corresponding mature protein. Thereby, these triplets may not function as initiation or termination codons, instead they may be translated into amino-acids. Even if these observations led us to think that all protein-coding genes start with the canonical initiation codon ATG and terminate with the ochre TAA or the amber TAG stop codons (Table 3), future functional studies should be conducted to validate these results. We also provide the detailed codon usage information in Table 5.

**Table 5:** Codon usage of the protein-coding genes in the mitochondrial genome of *S*. *lycopersici* CIDEFI-216.

Codon	Amino acid	Percentage	Frequency	Codon	Amino acid	Percentage	Frequency
GCA	Ala	33.7	179	CTT	Leu	15.1	215
GCC	Ala	8.5	45	TTA	Leu	60.6	865
GCG	Ala	5.6	30	TTG	Leu	8.0	114
GCT	Ala	52.2	277	AAA	Lys	85.4	653
AGA	Arg	68.7	266	AAG	Lys	14.6	112
AGG	Arg	9.8	38	ATG	Met	100	215
CGA	Arg	3.6	14	TTC	Phe	26.4	204
CGC	Arg	1.8	7	TTT	Phe	73.6	568
CGG	Arg	2.6	10	CCA	Pro	21.8	81
CGT	Arg	13.4	52	CCC	Pro	8.4	31
AAC	Asn	24.9	175	CCG	Pro	11.9	44
AAT	Asn	75.1	527	CCT	Pro	58.0	215
GAC	Asp	18.6	77	AGC	Ser	7.3	70
GAT	Asp	81.4	336	AGT	Ser	29.3	282
TGC	Cys	13.0	18	TCA	Ser	23.0	222
TGT	Cys	87.0	120	TCC	Ser	4.9	47
CAA	Gln	79.9	226	TCG	Ser	2.8	27
CAG	Gln	20.1	57	TCT	Ser	32.8	316
GAA	Glu	74.0	333	ACA	Thr	39.2	235
GAG	Glu	26.0	117	ACC	Thr	7.2	43
GGA	Gly	33.7	207	ACG	Thr	5.8	35
GGC	Gly	4.2	26	ACT	Thr	47.8	287
GGG	Gly	11.9	73	TGA	Trp	72.8	99
GGT	Gly	50.2	309	TGG	Trp	27.2	37
CAC	His	27.4	65	TAC	Tyr	27.1	155
CAT	His	72.6	172	TAT	Tyr	72.9	416
ATA	Ile	47.8	506	GTA	Val	43.2	275
ATC	Ile	9.8	104	GTC	Val	3.9	25
ATT	Ile	42.3	448	GTG	Val	10.2	65
CTA	Leu	12.4	177	GTT	Val	42.7	272
CTC	Leu	1.3	19	TAA	*	72.2	26
CTG	Leu	2.7	38	TAG	*	27.8	10

* Stop codón

### Repetitive elements accounted for about 5.6% of the mitochondrial genome

**Fig 4:**
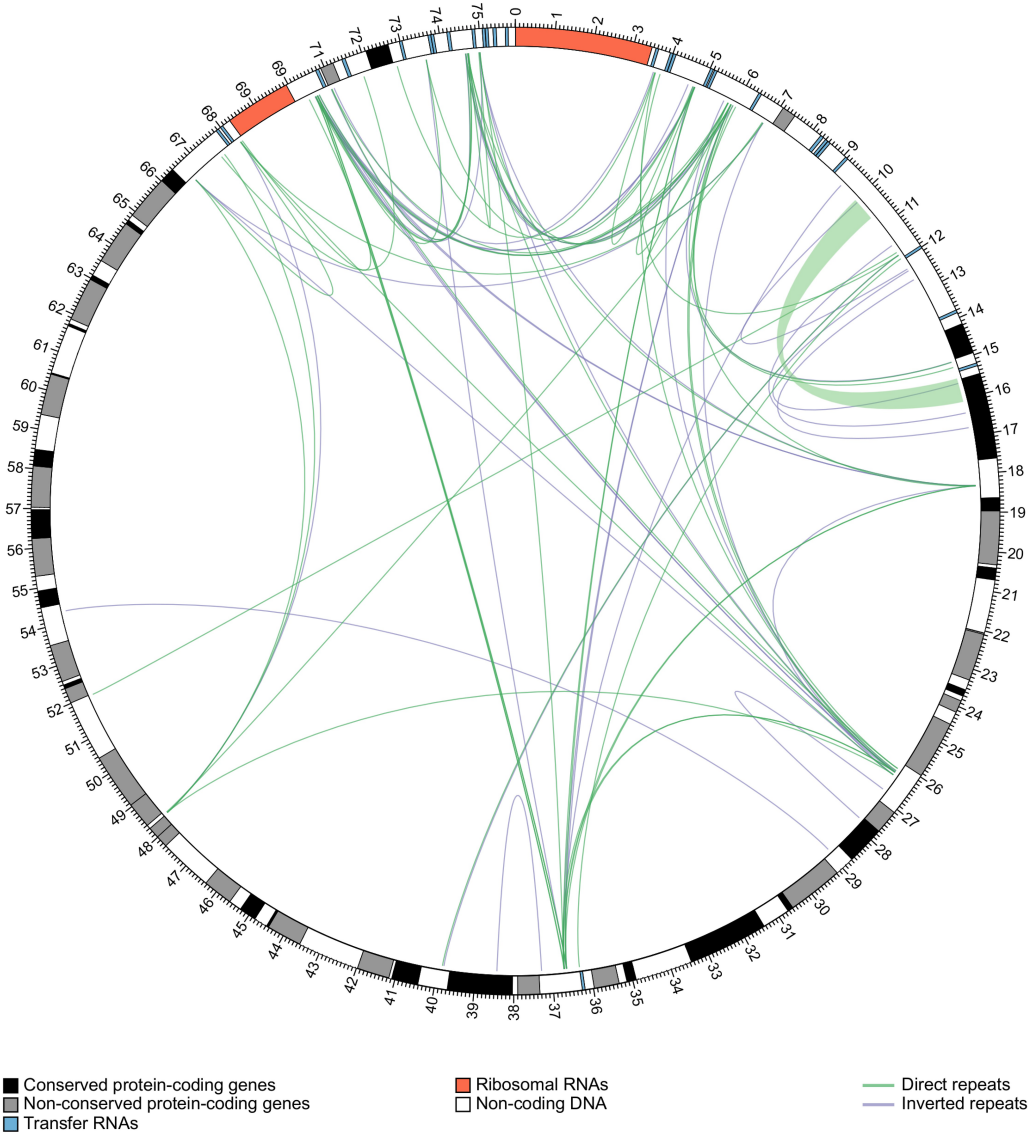
Map of the repetitive sequences in the mitochondrial genome of *S*. *lycopersici* CIDEFI-216. Lines connect repetitive sequences of at least 30 nucleotides in length and with a minimum match identity of 80%. A green ribbon shows the large duplication. The detailed references are given in the figure. Repetitive sequences were identified using the Vmatch software. The physical map was created with Circos.

**Fig 5:**
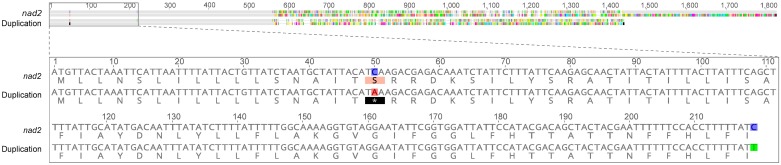
Sequence alignment between *nad2* and the large duplicated region found in the mitochondrial genome of *S*. *lycopersici*. The duplicated region has a length of 630 nucleotides, encompassing the first 590 nucleotides of *nad2*, albeit two SNPs (S17Stop and I219I) (See Fig 4). Nucleotide and amino-acid discrepancies are highlighted with colors. The nucleotide sequences were aligned using ClustalW with the default settings and were automatically translated using the translation Table 4 (The Mold, Protozoan, and Coelenterate Mitochondrial Code and the Mycoplasma/Spiroplasma Code) in Geneious 9.1.2.

One of the most notable features of the mitochondrial genome of *S*. *lycopersici* CIDEFI-216 is the abundance of both direct and palindromic repetitive sequences. In this regard, we found 84 repetitive elements larger than 30 nucleotides, which accounted for about 5.6% of the mitochondrial genome and exhibited an overall GC content of 25.1%. These repetitive sequences were unevenly distributed mostly throughout intergenic regions of the mitochondrial genome and showed a notable preference for some regions, among the most prominent ones were those located upstream and downstream the *rns* and *rnl*, between *orf185* and *orf210* and downstream *rps3* genes (Fig 4). We also observed the occurrence of a large duplicated region that contains a partial sequence of *nad2* (Fig 4, green ribbon). This duplication is 1332 nucleotides long and their first 558 nucleotides have a 99.6% pairwise identity with the first third of *nad2*, however there is a nonsense mutation in the 17th codon which results in the conversion of a Serine into a stop codon (S17Stop). Within this partial sequence, there is also one more single nucleotide polymorphism that may result in a silent mutation (I219I). The sequence downstream nucleotide 558 had no homology to any known protein (Fig 5). Probably, selective pressure might have generated a premature stop-codon within the gene arisen from the partial duplication of *nad2*, which might avoid expression of such a large non-functional protein, a process that may be rather expensive and yet unaffordable in terms of cellular energy.

Although they have no obvious function, repetitive sequences may play a role in the dynamics and evolution of mitochondrial genomes. Indeed, it is accepted that repetitive sequences are frequently involved in recombinations events that may not only lead to changes in gene order and orientation, but also to the generation of novel genes [84, 85]. Besides their intriguing origin and function (if any), mitochondrial repetitive sequences represent attractive molecular markers to study populations, considering that they can be easily amplified because of their high copy number.

### Lack of synteny among mitochondrial genomes of four representatives of Pleosporales

**Fig 6:**
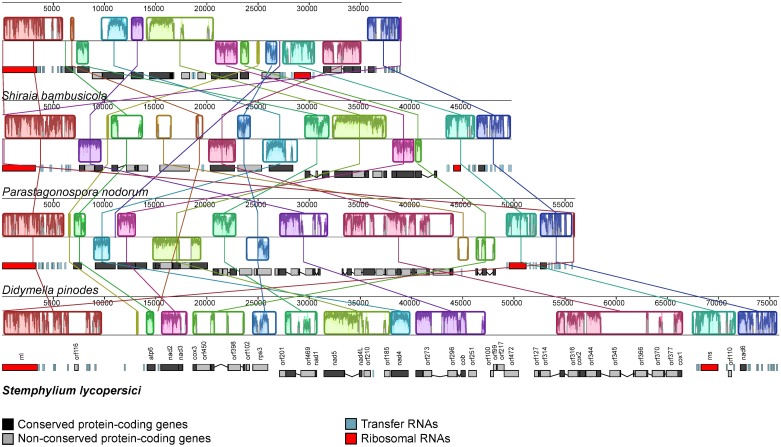
Whole mitochondrial alignments of four Pleosporales species. Gene order and orientation among mitochondrial genomes of the Pleosporales species *S*. *lycopersici* (KX453765), *D*. *pinodes* (KT946597), *P*. *nodorum* (EU053989) and *S*. *bambusicola* (KM382246). Locally collinear blocks are depicted by the same colors. The plot inside of them indicates the level of sequence similarity. The ruler above each genome represents the nucleotide positions. The detailed references are given in the figure. The mitochondrial genomes were arbitrary linearized starting at *rnl* and were aligned using Mauve with the default settings. The backbone output was then modified with Inkscape 0.91.

The alignment of the mitochondrial genomes of *S*. *lycopersici* (KX453765), *D*. *pinodes* (KT946597), *P*. *nodorum* [42] and *S*. *bambusicola* [43] revealed a remarkable variation in gene order and orientation (Fig 6). However, there are some conserved arrangements, like *nad2*-*nad3* and *cox1*-*cox2* gene pairs, which are next to each other, or even fused. Also, *nad4L* is next to *nad5*, though in *P*. *nodorum* [42] a non-conserved ORF between them was found. Similarly, *nad6* is nested between *rns* and *rnl* in a region rich in tRNA genes, but a non-conserved ORF is placed between *rns* and *nad6* in *D*. *pinodes* (KT946597) and *P*. *nodorum* [42]. In *S*. *bambusicola*, apart from *nad6*, *cox1* and *cox2* are also nested between *rns* and *rnl* [43]. It is also interesting the large difference in mitochondrial genomes sizes within the studied Pleosporales. In view of the multiple alignment, it seems that the mitochondrial genome expansion is related to the presence of non-conserved ORFs, introns and repetitive elements, which, in turn, are particularly common within intergenic regions, as it was shown in the mitochondrial genome of *S*. *lycopersici* CIDEFI-216 (Fig 4).

### Phylogenetic analysis of Pezizomycotina

**Fig 7:**
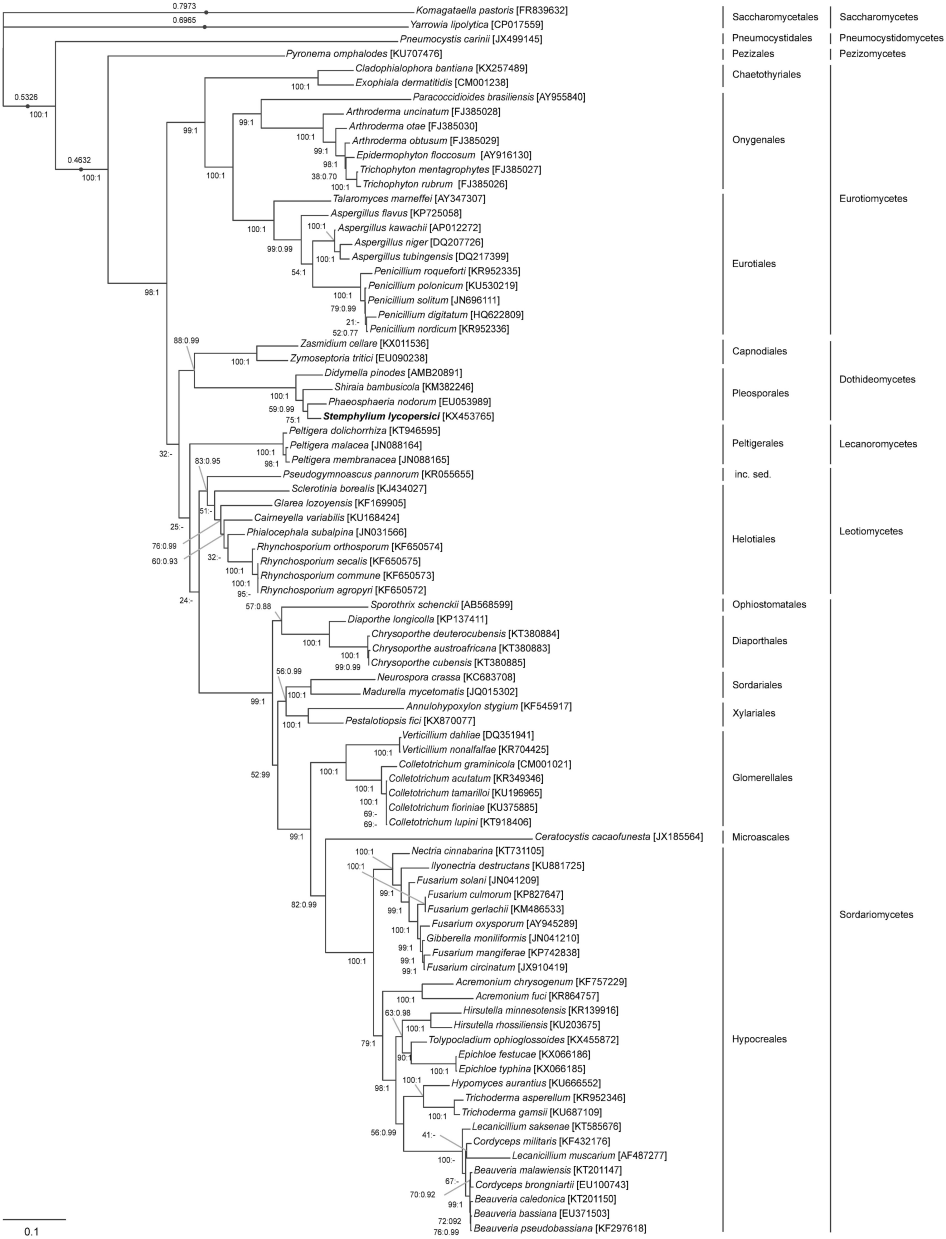
Phylogram of Pezizomycotina inferred from the concatenated sequences of 12 mitochondrial-encoded proteins. The data-set included the concatenated amino-acid sequences predicted from the mitochondrial-encoded *atp6*, *cob*, *cox1*, *cox2*, *cox3*, *nad1*, *nad2*, *nad3*, *nad4*, *nad4L*, *nad5* and *nad6*. Taxon sampling comprised 82 different species of Pezizomycotina, as well as *P*. *carinii* (Taphrinomycotina), *Y*. *lipolytica* and *K*. *pastoris* (Saccharomycotina) which were used as outgroups. GenBank accession numbers of the mitochondrial genomes are given in brackets. The corresponding families and orders are indicated at the right side of the figure. The maximum likelihood tree topology is shown (-lnL = -74201.930323). Numbers at the nodes represents maximum likelihood bootstrap support values (as a percentage of 1000 replicates) and Bayesian posterior probabilities, respectively. A hyphen (-) is used to indicate when a particular node was not recovered in the Bayesian inference. Branches with a dot represent branches whose lengths are not to scale and their actual lengths are indicated by the values placed above them. The scale bar represents the number of changes per sites.

Pezizomycotina is the largest subphylum of Ascomycota that includes remarkably diverse species in terms of their nutritional strategy [86]. Therefore, we further studied the systematic of Pezizomycotina and assessed the relationship of *S*. *lycopersici* with other taxa by means of a phylogenetic analysis using 12 conserved genes of 82 representatives of this subphylum. The analysis also included as outgroups a representative of Taphrinomycotina and two representatives of Saccharomycotina. Both maximum likelihood approach and Bayesian inference led to similar tree-topologies, except for the lack of congruence of a few internal nodes (Fig 7).

The phylogenetic tree strongly supports (bootstrap ≥ 99% and posterior probabilities = 1) that Pezizomycotina is a monophyletic subphylum that include the clade Leotiomyceta, Pezizomycetes (specifically its order Pezizales), Eurotiomycetes and its orders (Chaetothyriales, Onygenales and Eurotiales), Lecanoromycetes and its order (Peltigerales) and Sordariomycetes and its orders (Ophiostomatales, Diaporthales, Sordariales, Xylariales, Glomerellales and Microascales). Also, the class Dothideomycetes was resolved as monophyletic, albeit with a moderate support (bootstrap = 88%) in the maximum likelihood approach but with a strong support (posterior probabilities = 0.99) in the Bayesian inference. Nevertheless, both Capnodiales and Pleosporales were clearly shown to be monophyletic (bootstrap = 1 and posterior probabilities = 1). Similarly, the class Lecanoromycetes was resolved as monophyletic, but with lower support (bootstrap = 83% and posterior probabilities = 0.95), however members of the orders Helotiales and Leotiomycetes *inc*. *sed*. remained unsolved. The results described above are in accordance with the findings of Spatafora et. al, who reconstructed the phylogeny of Pezizomycotina based on five nuclear loci widely used in fungal taxonomy, namely: small subunit ribosomal DNA (SSU rDNA), long subunit ribosomal DNA (LSU rDNA), DNA-directed RNA polymerase II largest subunit (RPB1), DNA-directed RNA polymerase II second largest subunit (RPB2) and elongation factor 1-alpha (EF-1a) [86]. However, in our analysis members of Pezizomycotina are grouped together with the representative of Taphrinomycotina in a well-supported clade (bootstrap = 100% and posterior probabilities = 1), unlike most published data that support that Pezizomycotina and Saccharomycotina form a monophyletic group [86–89]. We argued that the observed difference might be due to some artifact of our phylogenetic reconstruction, particularly, by the Long Branch Attraction (LBA) effect, which could be explained by the clustering of fast-evolving linages irrespective of their evolutionary relationships. As a matter of fact, mitochondrial data of some Taphrinomycotina and Saccharomycotina species are known to be subject to LBA artifacts [90, 91]. Also, it is worth mentioning that *K*. *pastoris* and *Y*. *lipolytica* (both Saccharomycotina) and *P*. *carinii* (Taphrinomycotina) have a particular characteristic that distinguish them from some of other taxa of their subphyla, which is the atypical presence of *atp6*, *cob*, *cox1*, *cox2*, *cox3*, *nad1*, *nad2*, *nad3*, *nad4*, *nad4L*, *nad5* and *nad6* genes within their mitochondrial genomes [91–93], a feature that was, in fact, used as a criterion for taxon sampling.

Regarding the relationships among the taxa belonging to Pleosporales, we found that *S*. *lycopersici* (Pleosporaceae) was clustered together with *P*. *nodorum* (Phaeosphaeriaceae) in a moderate-supported group (bootstrap = 75% and posterior probabilities = 1) with *S*. *bambusicola* (Shiraiaceae) as sister taxon, which were also in a clade that was separated from *D*. *pinodes* (Didymellaceae), though with low support values. However, it is not our intention to discuss the taxonomical relationship of Shiraiaceae within Pleosporales due to the lack of exhaustive molecular phylogenetic studies including this family. Finally, it is important to point out that no correlation was found between the phylogenetic grouping of the Pleosporales representatives and the length of their mitochondrial genomes (Figs 6 and 7).

## Conclusions

The mitochondrial genome of *S*. *lycopersici* CIDEFI-216 harbors 12 genes encoding proteins involved in the oxidative phosphorylation and electron transport systems (*atp6*, *cob*, *cox1*, *cox2*, *cox3*, *nad1*, *nad2*, *nad3*, *nad4*, *nad4L*, *nad5* and *nad6*). *Atp8* and *atp9*, two other genes widely conserved among mitochondrial genomes of filamentous ascomycetes, where found in the nuclear genome. The mitochondrial genome of *S*. *lycopersici* CIDEFI-216 displays an unusual gene structure around cytochrome-c oxidase subunits 1 and 2, which led to a single continuous ORF encoding both subunits, as occurs in other Pleosporales species and distantly-related protozoan taxa. The mitochondrial genome of *S*. *lycopersici* CIDEFI-216 also has a freestanding *rps3* gene and 13 genes whose hypothetical proteins possess at least one recognizable homing endonuclease domain. The analysis of such a limited number of samples raises the question if such features could be considered hallmarks of Pleosporales, which will be solved soon, in view of the increased rate of sequenced fungal genomes. The comparison of gene order and orientation in *S*. *lycopersici* and its close relatives in the order Pleosporales revealed the plasticity of their mitochondrial genomes, which seems to be mediated in large part by the action of repetitive elements and homing endonucleases. Finally, it was further proved the utility of fungal mitochondrial genomes in comparative and phylogenetic studies and highlighted their potential for population studies.

## Supporting information

S1 Fig Sequence alignments of the nuclear-encoded atp8 and atp9 of *S*. *lycopersici* CIDEFI-216 with their mitochondrial-encoded homologues in *Z*. *cellare* and *Z*. *tritici*. (a) Sequence alignment of *atp8*. (b) Sequence alignment of *atp9*. Nucleotide and amino-acid identities are highlighted with colors. The nucleotide sequences were aligned using ClustalW with the default settings and were automatically translated using the translation Table 4 (The Mold, Protozoan, and Coelenterate Mitochondrial Code and the Mycoplasma/Spiroplasma Code) in Geneious 9.1.2. In addition, for the particular case of *S*. *lycopersici*, the automated translation was also carried out using the translation Table 1 (Standard), achieving the same results.
(EPS)

S2 Fig Detailed sequence alignment of COX1 and COX2 with two different gene configurations. Detailed sequence alignment of the schematic sequence alignment presented in Fig 2.
(EPS)

S3 Fig Schematic sequence alignment of RPS3 from Pleosporales species. Detailed sequence alignment of the schematic sequence alignment presented in Fig 3.
(EPS)

S1 Table Functional annotation of the non-conserved protein-coding genes of the mitochondrial genome of *Stemphylium lycopersici* CIDEFI-216. (XLSX)

S2 Table TargetP 1.1 predictions. (XLSX)
